# Potential candidates for liver resection in liver-confined advanced HCC: a Chinese multicenter observational study

**DOI:** 10.3389/fonc.2023.1170923

**Published:** 2023-06-26

**Authors:** Tingting Bai, Enxin Wang, Shoujie Zhao, Dandan Han, Yan Zhao, Hui Chen, Jun Zhu, Tenghui Han, Yang Bai, Yanju Lou, Yongchao Zhang, Man Yang, Luo Zuo, Jiahao Fan, Xing Chen, Jia Jia, Wenbin Wu, Weirong Ren, Yejing Zhu, Shouzheng Ma, Fenghua Xu, Yuxin Tang, Xilin Du, Junlong Zhao, Jing Li, Xingshun Qi, Ying Han, Dongfeng Chen, Lei Liu

**Affiliations:** ^1^ Department of Digestive Diseases, Daping Hospital, Army Medical University (Third Military Medical University), Chongqing, China; ^2^ Department of Digestive Diseases, Air Force Hospital of Western Theater Command, Chengdu, China; ^3^ Department of Digestive Diseases, Tangdu Hospital, Air Force Medical University (Fourth Military Medical University), Xi’an, China; ^4^ Department of General Surgery, The Air Force Hospital of Southern Theater Command, Guangzhou, China; ^5^ Department of Digestive Diseases, The First Affiliated Hospital of Xi’an Jiao Tong University, Xi’an, China; ^6^ Xijing Hospital of Digestive Diseases, Air Force Medical University (Fourth Military Medical University), Xian, China; ^7^ Department of General Surgery, The Southern Theater Air Force Hospital, Guangzhou, China; ^8^ Department of Internal Medicine, Air Force Medical University (Fourth Military Medical University), Xian, China; ^9^ Department of Neurosurgery, General Hospital of Northern Theater Command, Shenyang, China; ^10^ Department of Orthopedic Surgery, Air Force Hospital of Western Theater Command, Chengdu, China; ^11^ Department of Medical Affairs, Air Force Hospital of Western Theater Command, Chengdu, China; ^12^ Center for Digestive Disease, The Seventh Affiliated Hospital, Sun Yat-sen University, Shenzhen, China; ^13^ Department of Digestive Diseases, the Second Affiliated Hospital of Chengdu Medical College, Chengdu, China; ^14^ Department of Digestive Diseases, the Affiliated Hospital of Southwest Medical University, Luzhou, China; ^15^ Department of Oncology, Qingdao Women and Children’s Hospital, Qingdao, China; ^16^ Department of Emergency, Shaanxi Provincial People’s Hospital, Xi’an, China; ^17^ Department of Digestive Diseases, Xi’an First Hospital, Xi’an, China; ^18^ Department of Digestive Diseases, Sanmenxia Central Hospital, Henan University of Science and Technology, Sanmenxia, China; ^19^ Department of Surgery, Tangdu Hospital, Air Force Medical University (Fourth Military Medical University), Xi’an, China; ^20^ State Key Laboratory of Cancer Biology, Medical Genetics and Development Biology, Fourth Military Medical University, Xi’an, China; ^21^ Department of Digestive Diseases, Shanxi Bethune Hospital, Shanxi Academy of Medical Science, Tongji Shanxi Hospital, Third Hospital of Shanxi Medical University, Taiyuan, China; ^22^ Tongji Hospital, Tongji Medical College, Huazhong University of Science and Technology, Wuhan, China; ^23^ Department of Digestive Diseases, General Hospital of Northern Theater Command, Shenyang, China

**Keywords:** hepatocellular carcinoma, liver resection, performance status, overall survival, prognosis

## Abstract

**Background:**

Advanced hepatocellular carcinoma (HCC) is characterized as symptomatic tumors [performance status (PS) score of 1-2], vascular invasion and extrahepatic spread, but patients with PS1 alone may be eliminated from this stage. Although liver resection is used for liver-confined HCC, its role in patients with PS1 alone remains controversial. Therefore, we aimed to explore its application in such patients and identify potential candidates.

**Methods:**

Eligible liver-confined HCC patients undergoing liver resection were retrospectively screened in 15 Chinese tertiary hospitals, with limited tumor burden, liver function and PS scores. Cox-regression survival analysis was used to investigate the prognostic factors and develop a risk-scoring system, according to which patients were substratified using fitting curves and the predictive values of PS were explored in each stratification.

**Results:**

From January 2010 to October 2021, 1535 consecutive patients were selected. In the whole cohort, PS, AFP, tumor size and albumin were correlated with survival (adjusted P<0.05), based on which risk scores of every patient were calculated and ranged from 0 to 18. Fitting curve analysis demonstrated that the prognostic abilities of PS varied with risk scores and that the patients should be divided into three risk stratifications. Importantly, in the low-risk stratification, PS lost its prognostic value, and patients with PS1 alone achieved a satisfactory 5-year survival rate of 78.0%, which was comparable with that PS0 patients (84.6%).

**Conclusion:**

Selected patients with PS1 alone and an ideal baseline condition may benefit from liver resection and may migrate forward to BCLC stage A.

## Introduction

Hepatocellular carcinoma (HCC) is different from other solid tumors, and the prognosis of which is associated with not only tumor burden but also liver function and performance status (PS) (1). Considering these factors, many staging systems have been proposed for survival prediction in HCC (2–5). Widely adopted in clinical practice, the Barcelona Clinic Liver Cancer (BCLC) staging system has both excellent survival discrimination and available treatment allocation which divides HCC patients into five significantly different stages (6, 7). Unfortunately, many patients have been initially diagnosed with advanced HCC which is characterized by symptomatic tumors, vascular invasion and extrahepatic spread, thereby missing the opportunity for curative treatments (2). However, the survival benefit for advanced patients from systemic treatments recommended by the BCLC system is limited to a wide range of 6.4 to 19.2 months (8–12). Such a large survival variation results from the heterogeneity of advanced HCC.

Vascular invasion and extrahepatic spread are relevant to the malignancy of HCC, and the presence of which also indicates the deterioration of survival. Eastern Cooperative Oncology Group performance status score of 1 (ECOG-PS1) refers to patients not being able to engage in strenuous physical activity but able to carry out work of a light or sedentary nature, and it has been associated with survival (13). Previous studies have shown that the survival of patients with PS1 alone without vascular invasion or extrahepatic spread is significantly better than that of patients with vascular invasion or extrahepatic spread (7, 14). Remarkably, several studies have demonstrated that the survival of HCC patients with PS1 alone is different from that of other patients, and those patients should be migrated to the former stage (14, 15). Interestingly, another HCC staging system from East, the Hong Kong Liver Cancer (HKLC) Staging System suggests that the prognoses of patients with no or mild tumor-related symptoms (PS0 vs. PS1) are similar and have the same treatment allocation (3). Therefore, indiscriminately including patients with PS1 alone at an advanced stage may be challenging, and it should be explored whether they could migrate to the former stages and receive more aggressive treatments.

Liver resection is the first-line recommended treatment for early-stage diseases according to guidelines (2), and it is also adopted in real-world practice, especially for patients with PS1 alone (16–18). Previously, our team found advanced patients with PS1 alone and a single lesion had 1-, 3-, and 5-year survival rates of 83.2%, 60.8%, and 33.3%, respectively (6). Thus, we hypothesized that there is a certain subgroup of advanced patients with PS1 alone who may benefit from liver resection and even obtain comparable survival with PS0 patients.

The present study aimed to explore the predictive ability of PS in HCC patients treated by liver resection, identify the optimal candidates who would benefit from liver resection in PS1 alone, and propose a modification of the BCLC system.

## Materials and methods

### Study population and eligibility

**Figure 1:**
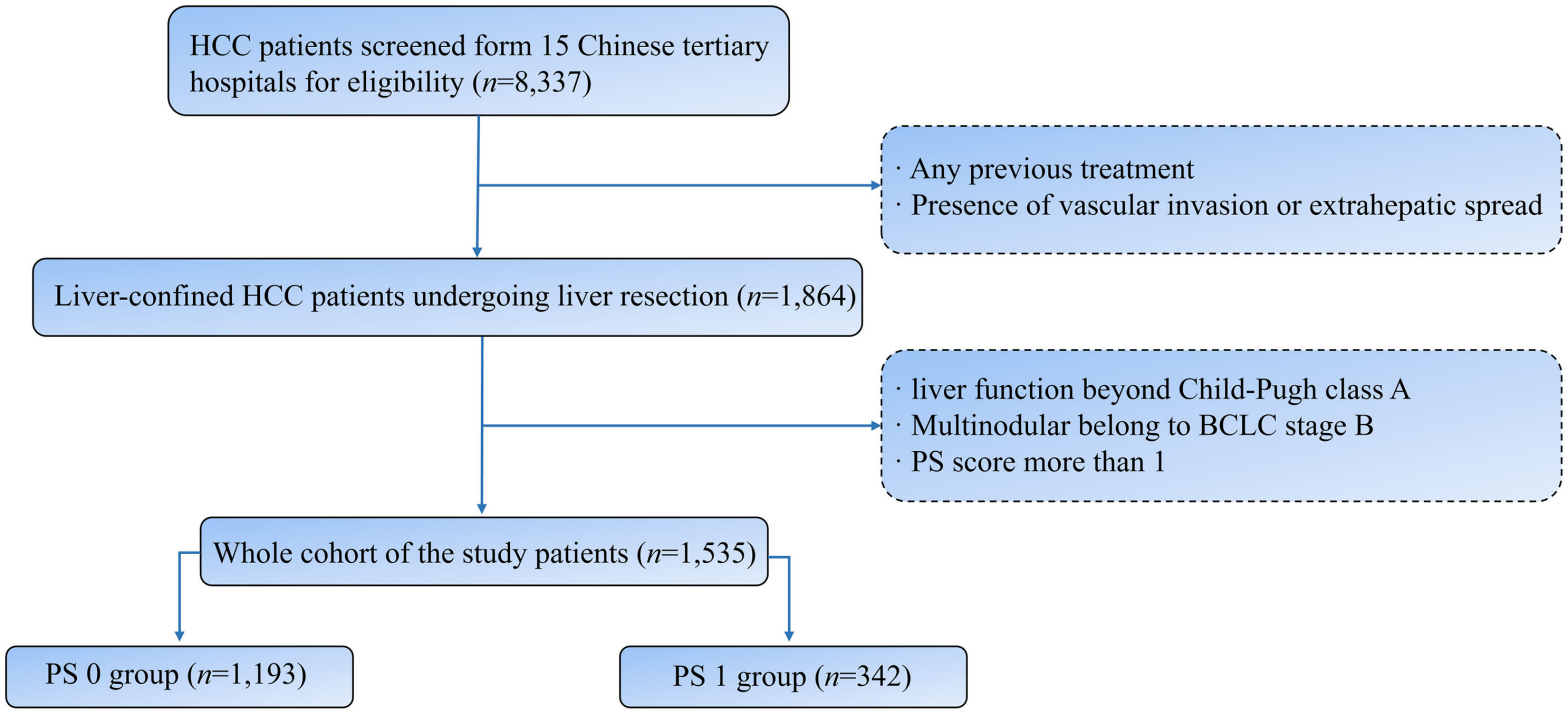
Flowchart of the patient selection. HCC, hepatocellular carcinoma; PS, performance status.

Form 15 Chinese tertiary hospitals, 8337 consecutive HCC patients undergoing liver resection were screened during the period from January 2010 to October 2021. The exclusion criteria were as follows: (I) Any previous treatment; (II) presence of vascular invasion or extrahepatic spread; (III) liver function beyond Child-Pugh class A; (IV) Multinodular belong to BCLC stage B; (V) PS score more than 1. Finally, 1535 patients were included in the study cohort (**Figure 1**). HCC was diagnosed by contrast-enhanced computed tomography (CT) and magnetic resonance imaging (MRI) according to the guidelines of the American Association for the Study of the Liver Disease or the European Association for the Study of Liver Disease (AASLD/EASL) (19, 20). Clinical, laboratory, and imaging data of registered patients were evaluated and collected by three independent clinicians from the database of every enrolled hospital.

### Treatment and follow-up

After general anesthesia administration, liver resection was performed through a right subcostal incision. The perihepatic ligaments and adhesion tissue were separated prior to abdominal exploration. Intraoperative ultrasound was used to assess the number, size, distribution, and invasion of adjacent structures of the liver tumor, as well as the relationship of the tumor to blood vessels, bile ducts, and other structures. Hepatic blood flow was blocked by applying the Pringle technique, and then hepatoduodenal ligament was clamped with a rubber tourniquet. Depending on the location, size, and number of tumors, patients underwent hepatic lobectomy, segmental hepatectomy, hemihepatectomy, or partial hepatectomy. Each procedure was performed in accordance with the standard approach recommended by the guidelines. All nodules were removed before being sent to the pathology department. Laboratory assessment and radiologic evaluation [contrast-enhanced computed tomography (CT) or magnetic resonance imaging (MRI)] were performed at one month after liver resection, every three months during the first year and every three to six months subsequently. Overall survival (OS) was defined as the time from the date of liver resection until death or the date of the last follow-up, and the last one occurred in October, 2021.

### Statistical analysis

Categorical variables are presented by frequencies and percentages, and continuous data are presented as the median with interquartile range (IQR). Overall survival (OS) was estimated using Kaplan–Meier curves and compared using the log-rank test. The median follow-up was estimated using Kaplan–Meier curves and compared using two independent sample nonparametric tests. The Cox proportional hazards regression model was used to analyze prognostic factors correlated with outcomes, where PS (PS0 vs. PS1) was used as a stratifying covariate. Considering the impacts of baseline characteristics on outcomes, we adjusted the difference in outcomes between PS stratification in multivariate regression models and propensity score matching (PSM) (21). First, every baseline variable was tested in univariate Cox regression models and then adjusted in multivariate models to identify the predictors for OS. Second, we performed 1:1 nearest neighbor PSM and set the caliper value to 0.2 to remove possible confounders and variables associated with survival (21). Baseline variables with significant differences between PS0 and PS1 patients, as well as variables associated with predictors of OS in univariate Cox regression models, were included in the PSM analysis. Finally, subgroup analysis was performed based on risk scores, which were calculated by variables’ prognostic values with the corresponding regression coefficients multiplied by 10 and rounded to the nearest integer. Moreover, the cutoff value of the subgroup was determined by fitting curves, and univariate and multivariate analyses were used to confirm the rationality of grouping. In addition, the sample size per subgroup met the 10 events per variable principle (10EVP) (22). Statistical analysis was conducted using SPSS software version 25.0 (SPSS Inc., Chicago, IL, USA) and R version 3.3.1 (R Foundation for Statistical Computing, Vienna, Austria).

## Results

### Patient characteristics

In total, 1535 eligible HCC patients were retrospectively selected in the present study, including 1193 (77.7%) patients with PS0 and 342 (22.3%) patients with PS1 (**Table 1**). The following characteristics were prevalent in the whole cohort: age less than 70 years (1416, 92.2%); male sex (1283, 83.6%); etiology of hepatitis B virus (HBV; 1426, 92.9%); Child-Pugh score of 5 (1392, 90.7%); α-fetoprotein (AFP) less than 400 ng/ml (1089, 70.9%); single tumor (1491, 97.1%); tumor size no more than 5 cm (951, 62.0%); and albumin more than 35 g/L (1426, 92.9%). In addition, patients with PS1 tended to have higher levels of aspartate aminotransferase (AST), white blood cells (WBC), platelets (PLT) and AFP as well as larger tumor sizes than those with PS0 (all P<0.05, **Table 1**).

**Table 1:** Baseline characteristics of the whole cohort.

Baseline characteristics	Total patients (n=1535)	Cohorts before PSM		Cohort of PS0 after PSM ^$^ (n=342)
		PS 0 (n=1193)	PS 1 (n=342)	
Age, <70/≥70, n (%)	1416 (92.2)/119 (7.8)	1102 (92.4)/91 (7.6)	314 (91.8)/28 (8.2)	310 (90.6)/32 (9.4)
Gender, male/female, n (%)	1283 (83.6)/252 (16.4)	995 (83.4)/198 (16.6)	288 (84.2)/54 (15.8)	295 (86.3)/47 (13.7)
Etiology, HBV (+)/HBV (-), n (%)	1426 (92.9)/109 (7.1)	1110 (93.0)/83 (7.0)	316 (92.4)/26 (7.6)	314 (91.8)/28 (8.2)
Child-pugh score, 5/6, n (%) ^*^	1392 (90.7)/143 (9.3)	1097 (92.0)/96 (7.9)	295 (86.3)/47 (13.7)	311 (90.9)/31 (9.1)
AFP, <400/≥400 ng/ml, n (%) ^*^	1089 (70.9)/446 (29.1)	858 (71.9)/335 (28.1)	231 (67.5)/111 (32.5)	231 (67.5)/111 (32.5)
Tumor number, 1/>1, n (%)	1491 (97.1)/44 (2.9)	1156 (96.9)/37 (3.10)	335 (98.0)/7 (2.0)	336 (98.2)/6 (1.8)
Tumor size, ≤5/>5 cm, n (%) ^*^	951 (62.0)/584 (38.0)	794 (66.5)/399 (33.5)	157 (45.9)/185 (54.1)	157 (45.9)/185 (54.1)
Albumin, >35/≤35 g/L, n (%)	1426 (92.9)/109 (7.1)	1106 (92.7)/87 (7.3)	320 (93.6)/22 (6.4)	320 (93.6)/22 (6.4)
TBIL, ≤17.1/>17.1 μmol/L, n (%)	974 (63.5)/561 (36.5)	749 (62.8)/444 (37.2)	225 (65.8)/117 (34.2)	214 (62.6)/128 (37.4)
AST, U/L, median (IQR) ^*^	35.0 (26.0-49.2)	34.0 (26.0-48.0)	37.0 (27.0-57.0)	34.0 (27.0-49.0)
ALT, U/L, median (IQR)	33.0 (23.0-51.0)	33.0 (23.0-51.0)	34.0 (24.0-54.0)	34.0 (24.0-52.0)
WBC, ×10^9^/L, median (IQR) ^*^	5.3 (4.2-6.6)	5.2 (4.1-6.4)	5.5 (4.4-7.0)	5.6 (4.6-6.8)
BUN, mmol/L, median (IQR)	5.4 (4.4-6.4)	5.3 (4.5-6.4)	5.3 (4.4-6.4)	5.4 (4.5-6.4)
PLT, ×10^9/^L, median (IQR)^*^	146.0 (107.0-188.0)	142.0 (104.0-183.0)	158.0 (114.7-207.8)	154.0 (118.0-194.0)
INR, median (IQR)	1.02 (0.97-1.07)	1.02 (0.97-1.07)	1.02 (0.95-1.07)	1.00 (0.97-1.02)

* Variables with significant difference between PS 0 and PS 1 cohorts (P<0.05).

^$^ Variables with significant difference between PS 0 and PS 1 cohorts (P<0.05) after a 1:1 propensity score matching (PSM; caliper value of 0.2).

HCC, hepatocellular carcinoma; PS, performance status; HBV, hepatitis B virus; AFP, alpha fetoprotein; TBIL, total bilirubin; AST, aspartate aminotransferase; ALT, alamine aminotransferase; WBC, white blood cell; BUN, blood urea nitrogen; PLT, platelet count; INR, international normalized ratio.

### Prognostic ability of the PS score

**Figure 2:**
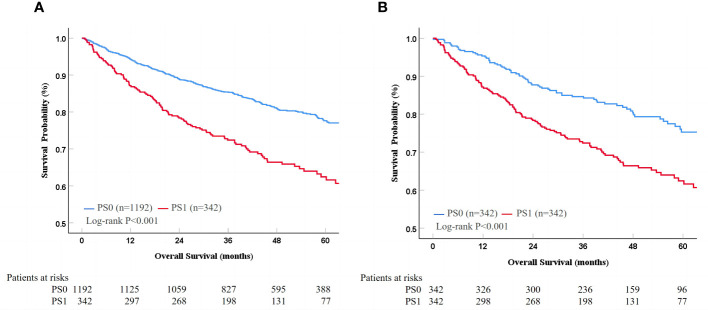
Survival difference between PS0 and PS1 patients in the whole **(A)** and PSM cohorts **(B)**. PS, performance status; PSM, propensity score matching.

The median follow-up duration was 54.4 (95% CI 52.8-6.0) months in the whole cohort. The median follow-up duration was 55.3 (95% CI 53.5-57.1) months in patients with PS0 and 54.4 (95% CI 52.8-55.9) month in patients with PS1 (P=0.395). During the period, a total of 354 (23.1%) patients died, and the mortality was 20.0% and 33.6% in the PS0 and PS1 groups, respectively. Kaplan-Meier curves demonstrated that patients with PS0 survived significantly longer than those with PS1 alone with 1-, 3-, and 5-year survival rates of 94.3%, 85.3%, and 77.4% in PS0 vs. 86.8%, 72.4%, and 61.6% in PS1, respectively (Log-rank P<0.001, **Figure 2A**). Univariate and multivariate analyses demonstrated that tumor size (adjusted HR 2.13, P<0.001), AFP (adjusted HR 1.67, P<0.001), albumin (adjusted HR 1.67, P<0.05) and PS (adjusted HR 0.61, P<0.001) were predictors of OS (**Table 2**). Considering that AST, WBC, PLT, AFP and tumor size were significantly different between patients with PS0 and PS1 as well as that AFP, tumor size and albumin were predictors of OS, we included these variables in the PSM analysis. Finally, there were 342 patients whose baseline variables and propensity scores were balanced in the two PS groups (all unadjusted P>0.05, **Table 1**). In the new cohort after PSM, the Kaplan-Meier curves showed that patients with PS0 had significantly better survival than those with PS1 alone (1-, 3-, and 5-year survival rates of 95.3%, 84.3%, and 75.3% in PS0 vs. 86.8%, 72.4%, and 61.6% in PS1, respectively; Log-rank P<0.001, **Figure 2B**). Similarly, PS remained a prognostic factor in the multivariate Cox-regression analysis model (adjusted HR 0.56 P<0.001, **Table 2**) and in an adjustment of the propensity score (adjusted HR 0.56, P=0.001).

**Table 2:** Univariate and multivariate analyses for predicting OS of the whole cohort.

Variables	Patients of the whole cohort				Patients after PSM			
	Univariate analyses		Multivariate analyses		Univariate analyses		Multivariate analyses	
	HR (95%CI)	P value	HR (95%CI)	P value	HR (95%CI)	P value	HR (95%CI)	P value
ECOG-PS score, Ref: PS score of 1	0.52 (0.41-0.65)	<0.001	0.61 (0.49-0.77)	<0.001	0.57 (0.43-0.77)	<0.001	0.56 (0.42-0.75)	<0.001
Age, Ref: Age <70 ng/mL	1.22 (0.85-1.74)	0.272	–	–	0.99 (0.60-1.63)	0.977	–	–
Genger, Ref: female	0.97 (0.73-1.28)	0.852	–	–	1.15 (0.75-1.76)	0.498	–	–
Etiology, Ref: HBV (-)	0.89 (0.61-1.30)	0.568	–	–	0.85 (0.52-1.39)	0.541	–	–
AFP, Ref: AFP <400 ng/mL	1.83 (1.48-2.26)	<0.001	1.67 (1.34-1.34)	<0.001	1.79 (1.34-2.39)	<0.001	1.62 (1.21-2.17)	<0.001
Tumor number, Ref: n=1	1.02 (0.54-1.92)	0.931	–	–	0.25 (0.03-1.85)	0.178	–	–
Tumor size, Ref: size ≤5 cm	2.43 (1.97-3.00)	<0.001	2.13 (1.72-2.64)	<0.001	2.52 (1.83-3.46)	<0.001	2.42 (1.76-3.33)	<0.001
Albumin, Ref: Albumin >35 g/L	1.66 (1.18-2.33)	0.003	1.67 (1.19-2.35)	0.003	1.28 (0.75-2.17)	0.351	–	–
TBIL, Ref: TBIL >17.1 μmol/L	1.02 (0.84-1.27)	0.838	–	–	0.99 (0.97-1.01)	0.600	–	–
AST, per 1U/L increase	1.00 (0.99-1.00)	0.692	–	–	1.00 (0.99-1.00)	0.913	–	–
ALT, per 1U/L increase	0.99 (0.99-1.00)	0.505	–	–	0.99 (0.99-1.00)	0.540	–	–
WBC, per 1×10^9^/L increase	1.01 (0.97-1.04)	0.526	–	–	1.01 (0.95-1.07)	0.574	–	–
BUN, per mmol/L increase	0.97 (0.91-1.02)	0.281	–	–	0.90 (0.82-0.99)	0.039	0.92 (0.85-1.01)	0.084
PLT, per 1×10^9^/L increase	1.00 (0.99-1.00)	0.849	–	–	1.00 (0.99-1.00)	0.654	–	–
INR, per 1 increase	1.10 (0.69-1.73)	0.681	–	–	0.94 (0.47-1.87)	0.681	–	–

After a propensity score matching (PSM), there were 342 patients in PS stratifications.

HR, hazard ratio; CI, confidence interval; ECOG, Eastern Cooperative Oncology Group; PS, performance status; HBV, hepatitis B virus; AFP, alpha-fetoprotein; TBIL, total bilirubin; AST, aspartate aminotransferase; ALT, alamine aminotransferase; WBC white blood cell; BUN, blood urea nitrogen; PLT, platelet count; INR, international normalized ratio.

### Risk stratification

**Figure 3:**
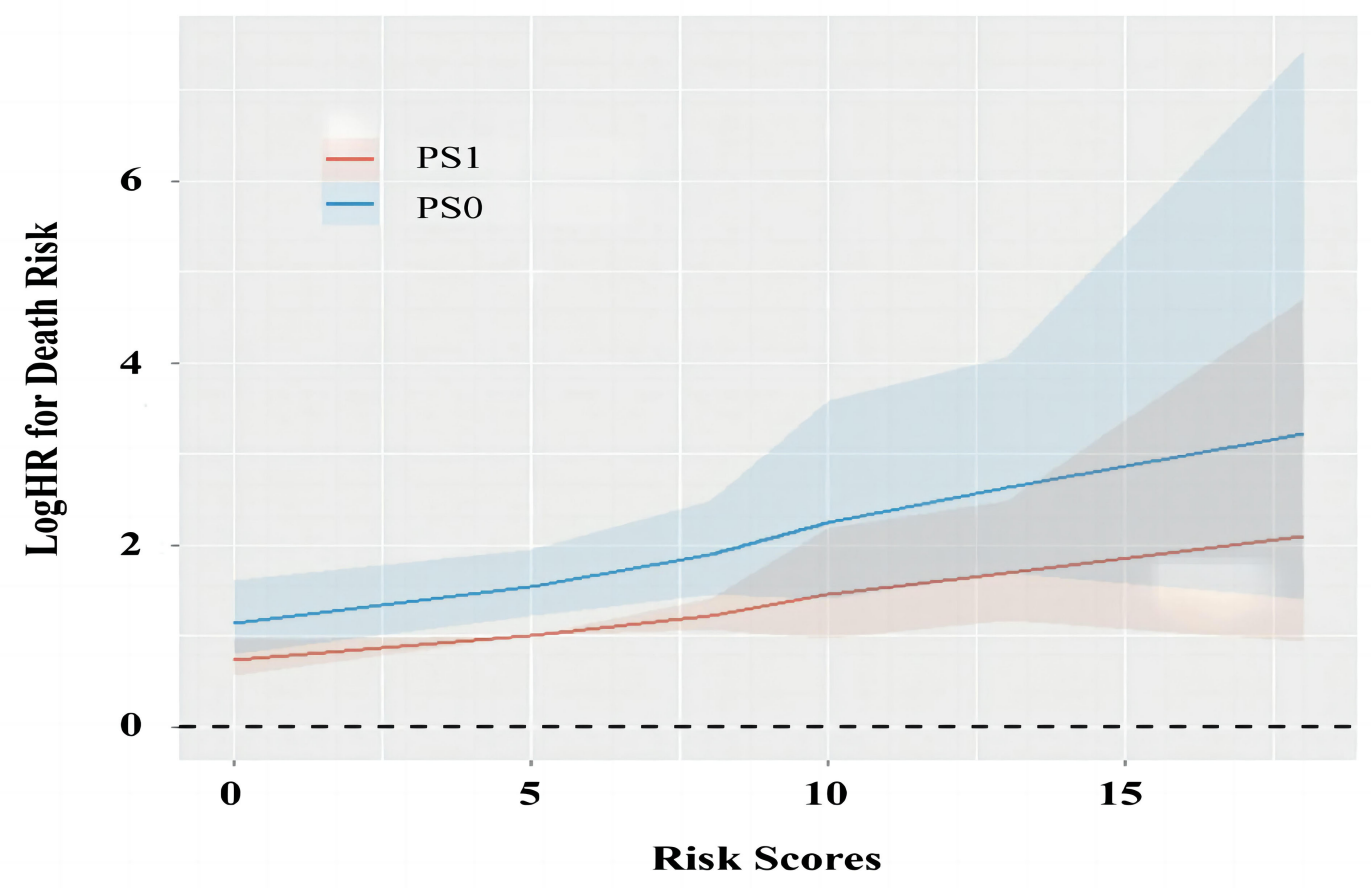
The change of prognostic values for PS0 and PS1 as the increase of risk scores. PS, performance status; HR, hazard ratio.

Patients were stratified according to the proposed risk scoring system, which was calculated by combining the prognostic factors of AFP, tumor size and albumin with their corresponding regression coefficients (AFP, 0.514; albumin, 0.518; and tumor size, 0.757) in the multivariate risk proportional regression model for the whole cohort. To simplify the calculation, the regression coefficients were multiplied by 10 and then rounded to the nearest integer. Thus, the risk score (R) was calculated as follow R = 5× (AFP: 0 if <400 ng/ml or 1 if ≥400 ng/ml) + 5× (albumin: 0 if >35 g/L or 1 if ≤35 g/L) + 8× (tumor size: 0 if ≤5 cm or 1 if > 5cm). There were six patient groups with risk scores of 0 (666, 43.4%), 5 (273, 17.8%), 8 (339, 22.1%), 10 (12, 0.8%), 13 (232, 15.1%), and 18 (13, 0.8%) (**Table 3**). In general, the hazards of death gradually improved as the risk scores increased regardless of the PS condition (**Table 3** and **Figure 3**). Furthermore, the fitting curves marginally crossed between 0 and 5 as well as near 8, which indicated that cutoff values were 0 and 8. According to these two cutoffs, we divided the patients into low- (R=0), medium- (R=5 or 8) and high-risk stratifications (R=10, 13 or 18) (**Table 3**).

**Table 3:** The prognostic value of PS in different risk scores.

Risk score	Patients (n)		Univariate analyses		Multivariate analyses		Variables included in multivariate analysis*
	PS 0	PS 1	HR (95%CI)	P value	HR (95%CI)	P value	
0	556	110	0.73 (0.44-1.22)	0.235	0.75 (0.45-1.25)	0.274	Age, Tumor number
5	228	45	0.45 (0.26-0.87)	0.016	0.50 (0.27-0.90)	0.022	PLT
8	234	105	0.49 (0.33-0.74)	0.001	0.50 (0.33-0.75)	0.001	Albumin
10	10	2	28.0 (0.01-39.0)	0.490	28.2 (0.00-40.1)	0.988	PLT, BUN, Age
13	156	76	0.69 (0.46-1.05)	0.084	0.70 (0.46-1.06)	0.091	PLT
18	9	4	0.69 (0.15-3.11)	0.633	0.37 (0.06-2.17)	0.275	AST

^*^ Variables with statistically significant differences in univariate analysis and included in multivariate analysis for adjustment.

HR, hazard ratio; CI, confidence interval; PS, performance status; AST, aspartate aminotransferase; PLT, platelet count; BUN, blood urea nitrogen.

### Prognostic ability of PS in low-risk stratification

**Figure 4:**
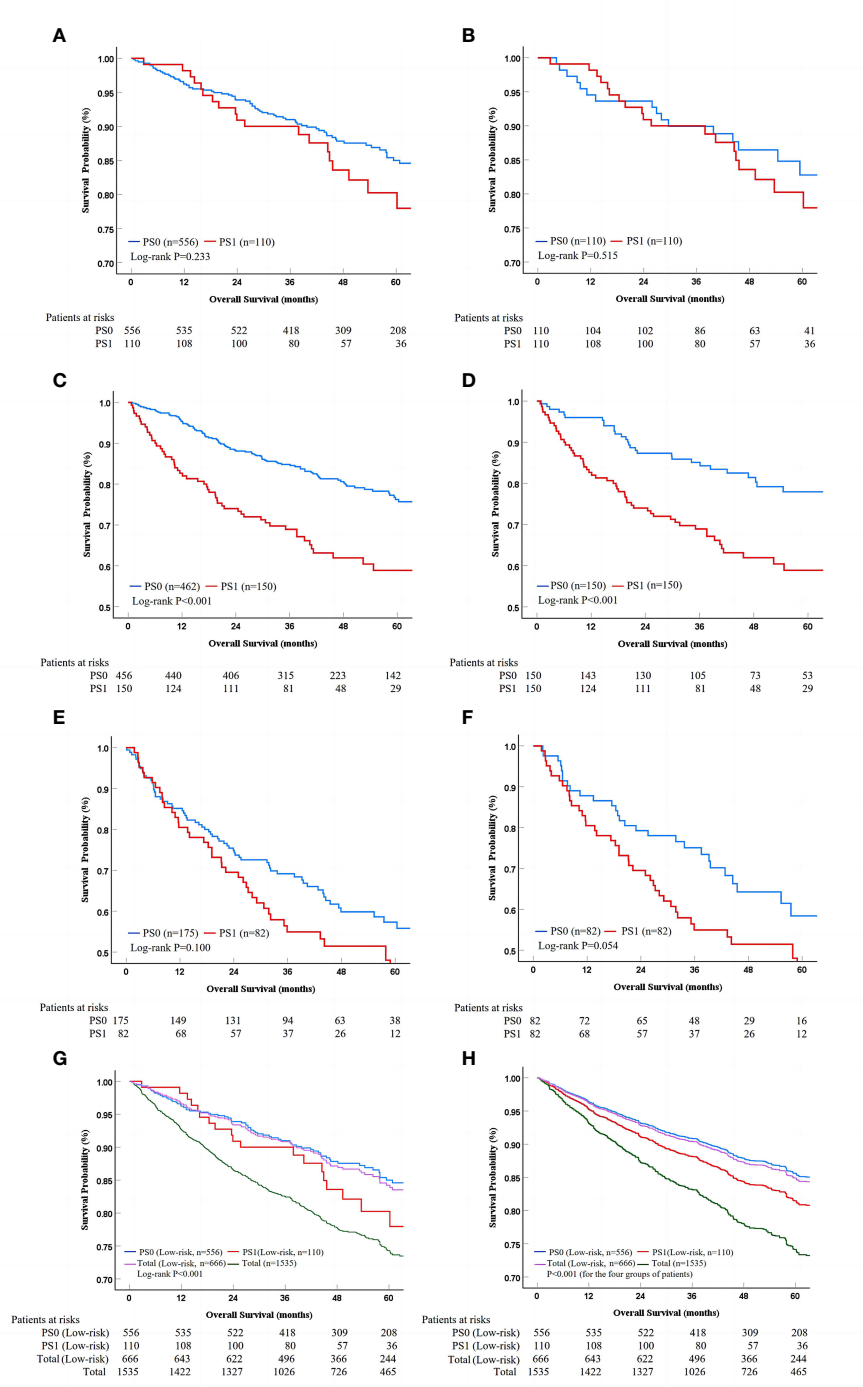
Survival analysis in different risk stratifications. PS, performance status; PSM, propensity score matching. The survival difference between PS0 and PS1 patients in the whole and PSM cohorts for low-risk **(A, B)**, medium-risk **(C, D)**, as well as high-risk stratification **(E, F)** according to Kaplan-Meier method. The low-risk patients could be identified from the whole cohort, while the PS score lost its prognostic value in univariate and multivariate analysis **(G, H)**.

For the low-risk stratification (R=0; AFP<400 ng/ml, albumin>35 g/L and tumor size ≤5cm), there were 556 (83.5%) patients with PS0 and 110 (16.5%) patients with PS1, and there were no differences in baseline characteristics in the two stratifications (all P>0.05, **Supplementary Table 1**). During a median follow-up duration of 56.1 (95% CI 53.9-58.3) months, patients with PS0 survived similarly to those with PS1 alone with 1-, 3-, and 5-year survival rates of 96.2%, 90.8%, and 84.6% in PS0 vs. 97.3%, 88.8%, and 78.0% in PS1, respectively (Log-rank P=0.233, **Figure 4A**). Multivariate analysis demonstrated that tumor number (adjusted HR 2.14, P<0.05) and age (adjusted HR 1.86, P<0.05) were independent predictors of OS, but PS (adjusted HR 0.73, P=0.242) was not an independent predictor of OS (**Table 4**). PSM analysis, including age and tumor number was conducted, which indicated that there were 110 patients with balanced baseline conditions in the two PS stratifications. After PSM, Kaplan-Meier curves showed that OS was not significantly different between patients with PS0 and PS1 stratifications with 1-, 3-, and 5-year survival rates were 93.6%, 88.9%, and 82.8% in PS0 vs. 97.3%, 88.8%, and 78.0% in PS1, respectively (Log-rank P=0.515, **Figure 4B**). In addition, the multivariate analysis with baseline characteristics (Model 2) and propensity score (Model 3) after PSM showed that PS was not a predictor of OS (all adjusted P>0.05, **Table 4**). In general, low-risk patients undergoing liver resection survived better than all the patients (Log-rank P<0.001, **Figure 4G**), and no survival difference was found between patients with PS0 and PS1 (Log-rank P=0.233, **Figures 4A, G**). More importantly, these findings were confirmed by multivariate analysis (adjusted P<0.001, **Figure 4H**).

**Table 4:** Prognostic abilities of PS in different risk stratifications.

Risk stratification	Univariate analysis		Multivariate analysis		Variables included in multivariate analysis*
	HR (95%CI)	P value	HR (95%CI)	P value	
Low-risk stratification					
Model 1	0.74 (0.44-1.22)	0.235	0.73 (0.44-1.22)	0.242	Age, Tumor number
Model 2	0.80 (0.41-1.56)	0.515	0.78 (0.39-1.53)	0.473	AST, ALT
Model 3	0.80 (0.41-1.56)	0.515	0.79 (0.40-1.55)	0.409	AST, ALT
Medium-risk stratification					
Model 1	0.48 (0.34-0.67)	<0.001	0.49 (0.35-0.68)	<0.001	AFP
Model 2	0.44 (0.28-0.70)	<0.001	0.46 (0.29-0.72)	0.001	AFP
Model 3	0.44 (0.28-0.70)	<0.001	0.45 (0.29-0.71)	0.001	AFP
High-risk stratification					
Model 1	0.72 (0.49-1.07)	0.102	0.71 (0.81-1.06)	0.096	PLT
Model 2	0.62 (0.39-1.01)	0.056	0.63 (0.39-1.03)	0.066	BUN, PLT
Model 3	0.62 (0.39-1.01)	0.056	0.63 (0.38-1.02)	0.062	BUN, PLT

^*^ Variables with statistically significant differences in univariate analysis and included in multivariate analysis for adjustment.

Model 1: multivariate analysis with baseline characteristics for original patients.

Model 2: multivariate analysis with baseline characteristics for patients after PSM.

Model 3: multivariate analysis with propensity score for patients after PSM.

HR, hazard ratio; CI, confidence interval; AST, aspartate aminotransferase; ALT, alamine aminotransferase; PLT, platelet count; BUN, blood urea nitrogen; AFP, alpha-fetoprotein.

### Prognostic ability of PS in medium- or high-risk stratification

For the medium- (R=5 or 8) and high-risk (R>8) stratifications, the baseline characteristics of the patients are shown and compared in **Supplementary Table 2**. Kaplan-Meier curves demonstrated that the patients with PS0 survived better than those with PS1 alone in the medium-risk stratification with the 1-, 3-, and 5-year survival rates of 95.2%, 84.5%, and 75.7% in PS0 vs. 82.0%, 67.1%, and 58.9% in PS1, respectively (Log-rank P<0.001, **Figure 4C**). However, patients with PS0 survived similarly to those with PS1 alone in the high-risk stratification with 1-, 3-, and 5-year survival rates of 84.6%, 68.4%, and 55.8% vs. 79.3%, 54.9%, and 40.5%, respectively (Log-rank P=0.100, **Figure 4E**). In PSM analysis, there were 150 and 82 patients in the medium- and high-risk stratifications with balanced propensity scores in the two PS groups (**Supplementary Table 2**). After PSM, Kaplan-Meier curves showed that patients with PS0 survived better than those with PS1 alone in the medium-risk stratification (Log-rank P<0.001, **Figure 4D**), but that they survived similarly in the high-risk stratification (Log-rank P=0.054, **Figure 4F**). Additionally, the multivariate analysis of three Models showed that PS was an independent predictor of OS in the medium-risk stratification (all adjusted P<0.05); on opposite, PS lost its prognostic value in the high-risk stratification (all adjusted P>0.05) (**Table 4**). Thus, these findings indicated that PS had significant prognostic ability only in the medium-risk stratification.

## Discussion

The present multicenter observational study demonstrated that PS remained an independent predictor of survival in patients with liver-confined HCC undergoing liver resection. Furthermore, AFP, albumin and tumor burden were combined to identify the patients who may benefit from liver resection and may be moved forward to BCLC stage A from advanced-HCC patients with PS1 alone. Compared to previous studies (14, 16, 23–25), the advantages of the present study included a large sample size specially focused on advanced HCC patients with PS1 alone undergoing liver resection, the use of subgroup analysis to explore the possibility of liver resection in managing some advanced diseases, and expansion of the indication of liver resection based on clinical practice.

A previous study has shown that PS significantly correlates with tumoral and cirrhotic factors for HCC patients (25). Similarly, in the present study, patients with PS1 had significantly higher levels of AST, WBC, PLT, AFP as well as larger tumor sizes than those with PS0 (P<0.05). Additionally, both univariate and multivariate analyses revealed that PS was an independent prognostic factor of OS. This finding agreed with previous studies, supporting the advancement of patients with PS1 alone to an advanced stage in the BCLC staging system (14, 25). However, some studies have reported contrasting conclusions (26, 27). In the HKLC staging system, PS0 and PS1 are equivalent and should be assigned to the same treatments (3). As mentioned above, PS1 indicates the presence of mild tumor-related symptoms that are influenced by nontumoral-related factors and subjectivity of HCC patients during symptom description; therefore, it is not easy to distinguish PS0 and PS1 in clinical practice. More importantly, advanced HCC patients have high heterogeneity, similar to those with PS1 alone. Consequently, inappropriately dividing some patients with PS1 alone into an advanced stage, who should be defined as early stage, may frequently occur in the real world. Several studies have confirmed that advanced HCC patients can benefit from liver resection (16, 28, 29), but these studies rarely focus on PS1 alone. In the present study, AFP, tumor size and albumin were identified as independent prognostic factors, which was consistent with previous studies and indicated high heterogeneity in such patients (30–32). It is necessary to further stratify HCC patients of PS1 alone according to these factors to identify appropriate candidates for liver resection.

A previous study including 2, 381 HCC patients has shown that defining patients with PS1 alone as BCLC stage B would increase the prognostic ability of the BCLC staging system and, proposes that BCLC stage C may not identify patients homogeneous enough to be allocated to a single stage (14). Furthermore, dividing HCC patients with BCLC stage B into four substages of B1 to B4 may be more appropriate than allocating patients with PS1 alone into the B4 substage instead of BCLC stage C (15). In our subgroup analysis of patients with low-risk stratification (R = 0; AFP <400 ng/ml, albumin >35 g/L and tumor size ≤5 cm), there was no difference in OS between patients with PS 0 and PS1 alone. Additionally, those patients with PS1 alone showed a promising OS after liver resection with a five-year survival rate of 78.0%, which was better than all patients in an advanced stage receiving systemic treatments (8–12). Therefore, liver resection may be an effective treatment for some advanced HCC patients with PS1 alone; thus, patients with PS1 alone should not be identified as BCLC stage C because they would lose the chance of curative therapies. In fact, many advanced HCC patients with PS1 are being treated by aggressive therapies and achieve considerable survival in real-world practice (14, 33, 34). Among the various treatments in addition to liver resection, TACE is also used for the treatment of advanced HCC patients with PS1 alone (6, 35). Our previous study indicated that liver resection is superior to TACE in advanced patients with PS 1 alone and single tumor, indicating that TACE should be considered as an alternative (6). Consequently, we suggested that HCC patients with PS1 alone with preferable baseline characteristics should be migrated to BCLC stage A with a similar prognosis to patients in the “real BCLC stage A”.

In the medium-risk stratification, we found that PS was also significantly associated with liver function, tumor burden and survival, which was consistent with previous studies (13, 36). In the whole cohort, PS remained a predictor of survival, which may have been due to high proportion of medium-risk patients (approximately 50%). In contrast, in the high-risk stratification, PS lost its prognostic value, and patients with PS0 and PS1 had a poor OS (with a five-year survival rate of 55.8% vs. 40.5%) after liver resection. This was probably because HCC patients with poor baseline characteristics may benefit little from liver resection and the effects of PS on survival would be diluted by other baseline factors. An Italian study has demonstrated no significant prognostic ability of PS in advanced HCC patients receiving best supportive care, supporting our findings (14). In another study from Taipei reported that the baseline characteristics become poorer with the deterioration of PS, and that the prognostic value gradually disappear (25). In summary, advanced HCC patients with PS1 alone and a medium-high stratifications have poor survival after liver resection and should still follow the recommendations of the BCLC staging system. Conversely, for patients with PS1 alone and a low-risk stratification, a modification of the BCLC staging system in patient stratification and treatment allocation should be considered.

The present study had several limitations. First, due to the retrospective design of our study, the evaluation of PS was based on the clinical data record, which may have introduced some subjectivity and inevitable information biases. To control these biases, the assessment of PS was conducted by three independent experienced clinicians, and the clinicians discussed any disagreements, especially for patients with no or mild tumor-related symptoms (PS0 or PS1). Second, due to the decrease in sample size in every risk stratification, the statistical power may have been weakened in the subgroup analysis. Therefore, several methods were used to adjust the prognostic value of PS. Importantly, the sample size per subgroup met the 10EVP, which ensured the rationality and reliability of the subgroup analysis. Third, the risk scoring formula consisted of three independent prognostic factors (AFP, tumor size and albumin), but their interaction effects were ignored. However, when separately using the ALBI grade, tumor burden or AFP for risk stratifications, PS remained predictive in every subgroup, indicating that these factors should not be used to identify the target candidates (all P<0.05, **Supplementary Table 3**). Finally, considering that the main etiology of HCC in Chinese patients of the present study were HBV infection, while the main etiologies of HCC in western patients were HCV, NAFLD and alcohol, our findings didn’t favour BCLC system caution should be used when generalizing and applying our findings.

## Conclusions

PS is an independent predictor of survival for liver-confined HCC patients undergoing liver resection. Patients with PS1 alone and an ideal baseline condition may significantly benefit from liver resection and should be migrated to BCLC stage A. Future high-quality studies focusing on this subset of patients with prospective design and external validation, should be conducted.

## Data availability statement

The raw data supporting the conclusions of this article will be made available by the authors, without undue reservation.

## Ethics statement

The study protocol conformed to the ethical guidelines of the 1975 Declaration of Helsinki and was approved by the Ethics Committee of the Army Medical Center of PLA on human research (Ratification NO: 2022 (121)).

## Author contributions

Study concept and design (EXW, LL), acquisition of data (TTB, EXW, SJZ, DDH, YZ, HC, JZ, THH, YB, YJL, YCZ, MY, LZ, JHF, XC, JJ, WBW, WRR, YJZ, SZM, FHX, YXT, XLD, JLZ), analysis and interpretation of data (TTB, EXW, SJZ, LL), drafting of the manuscript (TTB, EXW, SJZ).

## References

[1] Wan G Gao F Chen J Li Y Geng M Sun L. Nomogram prediction of individual prognosis of patients with hepatocellular carcinoma. BMC Cancer (2017) 17(1):91. doi: 10.1186/s12885-017-3062-6 28143427 PMC5286659

[2] Reig M Forner A Rimola J Ferrer-Fàbrega J Burrel M Garcia-Criado Á. BCLC strategy for prognosis prediction and treatment recommendation: the 2022 update. J Hepatol (2022) 76(3):681–93. doi: 10.1016/j.jhep.2021.11.018 PMC8866082 34801630

[3] Yau T Tang VY Yao TJ Fan ST Lo CM Poon RT. Development of Hong Kong liver cancer staging system with treatment stratification for patients with hepatocellular carcinoma. Gastroenterology (2014) 146(7):1691–700.e3. doi: 10.1053/j.gastro.2014.02.032 24583061

[4] Marrero JA Fontana RJ Barrat A Askari F Conjeevaram HS Su GL. Prognosis of hepatocellular carcinoma: comparison of 7 staging systems in an American cohort. Hepatology (2005) 41(4):707–16. doi: 10.1002/hep.20636 15795889

[5] Hsu CY Huang YH Hsia CY Su CW Lin HC Loong CC. A new prognostic model for hepatocellular carcinoma based on total tumor volume: the Taipei integrated scoring system. J Hepatol (2010) 53(1):108–17. doi: 10.1016/j.jhep.2010.01.038 20451283

[6] Zhao S Zhang X Wang M Tan K Dou W Fan Q. Identifying optimal candidates for liver resection or transarterial chemoembolisation in patients with unresectable hepatocellular carcinoma. Ann Transl Med (2020) 8(9):586. doi: 10.21037/atm.2020.02.83 32566613 PMC7290527

[7] Golfieri R Bargellini I Spreafico C Trevisani F. Patients with Barcelona clinic liver cancer stages b and c hepatocellular carcinoma: time for a subclassification. Liver Cancer (2019) 8(2):78–91. doi: 10.1159/000489791 31019899 PMC6465743

[8] Mei K Qin S Chen Z Liu Y Wang L Zou J. Camrelizumab in combination with apatinib in second-line or above therapy for advanced primary liver cancer: cohort a report in a multicenter phase Ib/II trial. J Immunother Cancer (2021) 9(3). doi: 10.1136/jitc-2020-002191 PMC7986650 33741732

[9] Qin S Bi F Gu S Bai Y Chen Z Wang Z. Donafenib versus sorafenib in first-line treatment of unresectable or metastatic hepatocellular carcinoma: a randomized, open-label, parallel-controlled phase II-III trial. J Clin Oncol (2021) 39(27):3002–11. doi: 10.1200/jco.21.00163 PMC8445562 34185551

[10] Finn RS Qin S Ikeda M Galle PR Ducreux M Kim TY. Atezolizumab plus bevacizumab in unresectable hepatocellular carcinoma. N Engl J Med (2020) 382(20):1894–905. doi: 10.1056/NEJMoa1915745 32402160

[11] Llovet JM Ricci S Mazzaferro V Hilgard P Gane E Blanc JF. Sorafenib in advanced hepatocellular carcinoma. N Engl J Med (2008) 359(4):378–90. doi: 10.1056/NEJMoa0708857 18650514

[12] Kudo M Finn RS Qin S Han KH Ikeda K Piscaglia F. Lenvatinib versus sorafenib in first-line treatment of patients with unresectable hepatocellular carcinoma: a randomised phase 3 non-inferiority trial. Lancet (2018) 391(10126):1163–73. doi: 10.1016/s0140-6736(18)30207-1 29433850

[13] Orman ES Ghabril M Chalasani N. Poor performance status is associated with increased mortality in patients with cirrhosis. Clin Gastroenterol Hepatol (2016) 14(8):1189–1195.e1. doi: 10.1016/j.cgh.2016.03.036 27046483 PMC4955687

[14] Giannini EG Bucci L Garuti F Brunacci M Lenzi B Valente M. Patients with advanced hepatocellular carcinoma need a personalized management: a lesson from clinical practice. Hepatology (2018) 67(5):1784–96. doi: 10.1002/hep.29668 29159910

[15] Bolondi L Burroughs A Dufour JF Galle PR Mazzaferro V Piscaglia F. Heterogeneity of patients with intermediate (BCLC b) hepatocellular carcinoma: proposal for a subclassification to facilitate treatment decisions. Semin Liver Dis (2012) 32(4):348–59. doi: 10.1055/s-0032-1329906 23397536

[16] Famularo S Donadon M Cipriani F Giuliante F Ferri S Celsa C. Hepatectomy versus sorafenib in advanced nonmetastatic hepatocellular carcinoma: a real-life multicentric weighted comparison. Ann Surg (2022) 275(4):743–52. doi: 10.1097/sla.0000000000005373 35081572

[17] Liu YW Yong CC Lin CC Wang CC Chen CL Cheng YF. Liver resection of hepatocellular carcinoma within and beyond the Barcelona clinic liver cancer guideline recommendations: results from a high-volume liver surgery center in East Asia. J Surg Oncol (2020) 122(8):1587–94. doi: 10.1002/jso.26183 32815189

[18] Yamamoto M Kobayashi T Honmyo N Oshita A Abe T Kohashi T. Liver resection is associated with good outcomes for hepatocellular carcinoma patients beyond the Barcelona clinic liver cancer criteria: a multicenter study with the Hiroshima surgical study group of clinical oncology. Surgery (2022) 171(5):1303–10. doi: 10.1016/j.surg.2021.09.009 34756748

[19] Heimbach JK Kulik LM Finn RS Sirlin CB Abecassis MM Roberts LR. AASLD guidelines for the treatment of hepatocellular carcinoma. Hepatology (2018) 67(1):358–80. doi: 10.1002/hep.29086 28130846

[20] EASL clinical practice guidelines: management of hepatocellular carcinoma. J Hepatol (2018) 69(1):182–236. doi: 10.1016/j.jhep.2018.03.019 29628281

[21] Austin PC. An introduction to propensity score methods for reducing the effects of confounding in observational studies. Multivariate Behav Res (2011) 46(3):399–424. doi: 10.1080/00273171.2011.568786 21818162 PMC3144483

[22] Peduzzi P Concato J Feinstein A R Holford TR. Importance of events per independent variable in proportional hazards regression analysis. II. accuracy and precision of regression estimates. J Clin Epidemiol (1995) 48(12):1503–10. doi: 10.1016/0895-4356(95)00048-8 8543964

[23] Hyun MH Lee YS Kim JH Lee CU Jung YK Seo YS. Hepatic resection compared to chemoembolization in intermediate- to advanced-stage hepatocellular carcinoma: a meta-analysis of high-quality studies. Hepatology (2018) 68(3):977–93. doi: 10.1002/hep.29883 29543988

[24] Zhong JH Ke Y Gong WF Xiang BD Ma L Ye XP. Hepatic resection associated with good survival for selected patients with intermediate and advanced-stage hepatocellular carcinoma. Ann Surg (2014) 260(2):329–40. doi: 10.1097/sla.0000000000000236 24096763

[25] Hsu CY Lee YH Hsia CY Huang YH Su CW Lin HC. Performance status in patients with hepatocellular carcinoma: determinants, prognostic impact, and ability to improve the Barcelona clinic liver cancer system. Hepatology (2013) 57(1):112–9. doi: 10.1002/hep.25950 22806819

[26] Chien SC Chen CY Cheng PN Liu YS Cheng HC Chuang CH. Combined transarterial Embolization/Chemoembolization-based locoregional treatment with sorafenib prolongs the survival in patients with advanced hepatocellular carcinoma and preserved liver function: a propensity score matching study. Liver Cancer (2019) 8(3):186–202. doi: 10.1159/000489790 31192155 PMC6547299

[27] Wang Y Shen J Feng S Liang R Lai J Li D. Hepatic resection versus transarterial chemoembolization in infiltrative hepatocellular carcinoma: a multicenter study. J Gastroenterol Hepatol (2020) 35(12):2220–8. doi: 10.1111/jgh.15060 32246889

[28] Vitale A Burra P Frigo AC Trevisani F Farinati F Spolverato G. Survival benefit of liver resection for patients with hepatocellular carcinoma across different Barcelona clinic liver cancer stages: a multicentre study. J Hepatol (2015) 62(3):617–24. doi: 10.1016/j.jhep.2014.10.037 25450706

[29] Lee JM Jang BK Lee YJ Choi WY Choi SM Chung WJ. Survival outcomes of hepatic resection compared with transarterial chemoembolization or sorafenib for hepatocellular carcinoma with portal vein tumor thrombosis. Clin Mol Hepatol (2016) 22(1):160–7. doi: 10.3350/cmh.2016.22.1.160 PMC4825165 27044767

[30] Johnson PJ Berhane S Kagebayashi C Satomura S Teng M Reeves HL. Assessment of liver function in patients with hepatocellular carcinoma: a new evidence-based approach-the ALBI grade. J Clin Oncol (2015) 33(6):550–8. doi: 10.1200/jco.2014.57.9151 PMC4322258 25512453

[31] Liu C Yang H Feng Y Liu C Rui F Cao Y. A K-nearest neighbor model to predict early recurrence of hepatocellular carcinoma after resection. J Clin Transl Hepatol (2022) 10(4):600–7. doi: 10.14218/jcth.2021.00348 PMC9396318 36062279

[32] Sun J Li WG Wang Q He WP Wang HB Han P. Hepatic resection versus stereotactic body radiation therapy plus transhepatic arterial chemoembolization for Large hepatocellular carcinoma: a propensity score analysis. J Clin Transl Hepatol (2021) 9(5):672–81. doi: 10.14218/jcth.2020.00188 PMC8516846 34722182

[33] Aziz AO Omran D Nabeel MM Elbaz TM Abdelmaksoud AH Attar IE. Aggressive treatment of performance status 1 and 2 HCC patients significantly improves survival - an Egyptian retrospective cohort study of 524 cases. Asian Pac J Cancer Prev (2016) 17(5):2539–43. 27268626

[34] Hsu CY Liu PH Lee YH Hsia CY Huang YH Chiou YY. Aggressive therapeutic strategies improve the survival of hepatocellular carcinoma patients with performance status 1 or 2: a propensity score analysis. Ann Surg Oncol (2015) 22(4):1324–31. doi: 10.1245/s10434-014-4151-2 25326394

[35] Zhao S Dou W Fan Q Hu J Li H Zhang X. Identifying optimal candidates of transarterial chemoembolization (TACE) vs. sorafenib in patients with unresectable hepatocellular carcinoma. Ann Transl Med (2020) 8(9):587. doi: 10.21037/atm.2020.02.123 32566614 PMC7290559

[36] Nishikawa H Kita R Kimura T Ohara Y Sakamoto A Saito S. Clinical implication of performance status in patients with hepatocellular carcinoma complicating with cirrhosis. J Cancer (2015) 6(4):394–402. doi: 10.7150/jca.11212 25767611 PMC4349881

