# Supplementary material for: Potential candidates for liver resection in liver-confined advanced HCC: a Chinese multicenter observational study
Source: Front Oncol. 2023 Jun 26;13:1170923. doi: 10.3389/fonc.2023.1170923 (PMC10332657; doi:10.3389/fonc.2023.1170923)
Supplement: Supplementary file 1 [file Table_1.docx]

**Supplementary table 1. Baseline characteristics of low-risk cohort**

| **Baseline characteristics** | **Total patients (n=666)** | **Cohorts before PSM** | | **Cohort of PS0 after PSM ^$^**  **(n=110)** |
| --- | --- | --- | --- | --- |
|  |  | **PS 0 (n=556)** | **PS 1 (n=110)** |  |
| Age, <70 / ≥70, n (%) | 618 (92.8) / 48 (7.2) | 516 (92.8) / 40 (7.2) | 102 (92.7) / 8 (7.3) | 102 (92.7) / 8 (7.3) |
| Gender, male / female, n (%) | 565 (84.8) / 101 (15.2) | 470 (84.5) / 86 (15.5) | 95 (86.4) / 15 (13.6) | 96 (87.3) / 14 (12.7) |
| Etiology, HBV (+) / HBV (-)，n (%) | 621 (93.2) / 45 (6.8) | 516 (92.8) / 40 (7.2) | 105 (95.5) / 5 (4.5) | 103 (93.6) / 7 (6.4) |
| Child-pugh score, 5 / 6, n (%) | 640 (96.1) / 26 (3.9) | 532 (95.7) / 24 (4.3) | 108 (98.2) / 2 (1.2) | 104 (94.5) / 6 (5.5) |
| AFP, <400 / ≥400 ng/ml, n (%) | 666 (100.0) / 0 (0.0) | 556 (100.0) / 0 (0.0) | 110 (100.0) / 0 (0.0) | 110 (100.0) / 0 (0.0) |
| Tumor number, 1 / >1, n (%) | 638 (95.8) / 28 (4.2) | 532 (95.7) / 24 (4.3) | 106 (96.4) / 4 (3.6) | 106 (96.4) / 4 (3.6) |
| Tumor size, ≤5 / >5 cm, n (%) | 666 (100.0) / 0 (0.0) | 556 (100.0) / 0 (0.0) | 110 (100.0) / 0 (0.0) | 110 (100.0) / 0 (0.0) |
| Albumin, >35 / ≤35 g/L, n (%) | 666 (100.0) / 0 (0.0) | 556 (100.0) / 0 (0.0) | 110 (100.0) / 0 (0.0) | 110 (100.0) / 0 (0.0) |
| TBIL, ≤17.1 / >17.1 μmol/L, n (%) | 424 (63.7) / 242 (36.3) | 353 (63.5) / 203 (36.5) | 71 (64.5) / 39 (35.5) | 64 (52.8) / 46 (41.8) |
| AST, U/L, median (IQR) | 31.0 (24.0-42.4) | 34.0 (26.0-48.0) | 37.0 (27.0-57.0) | 34.0 (26.0-48.0) |
| ALT, U/L, median (IQR) | 33.0 (22.0-47.6) | 33.0 (23.0-51.0) | 34.0 (24.0-54.0) | 33.0 (23.0-51.0) |
| WBC, ×10^9^ /L, median (IQR) | 5.1 (4.1-6.2) | 5.2 (4.1-6.4) | 5.5 (4.4-7.0) | 5.2 (4.1-6.4) |
| BUN, mmol/L, median (IQR) | 5.4 (4.5-6.4) | 5.3 (4.5-6.4) | 5.3 (4.4-6.4) | 5.3 (4.5-6.4) |
| PLT, ×10^9^ /L, median (IQR) ^$^ | 132.0 (97.8-170.0) | 142.0 (104.0-183.0) | 158.0 (114.7-207.8) | 142.0 (104.0-183.0) |
| INR, median (IQR) | 1.02 (0.96-1.07) | 1.02 (0.97-1.07) | 1.02 (0.95-1.07) | 1.02 (0.97-1.07) |
| *Variables with significant difference between PS 0 and PS 1 cohorts (P＜0.05).  ^$^Variables with significant difference between PS 0 and PS 1 cohorts (P＜0.05) after a 1:1 propensity score matching (PSM; caliper value of 0.2).  Abbreviations: HCC, hepatocellular carcinoma; PS, performance status; HBV, hepatitis B virus; AFP, alpha fetoprotein; TBIL, total bilirubin; AST, aspartate aminotransferase; ALT, alamine aminotransferase; WBC, white blood cell; BUN, blood urea nitrogen; PLT, platelet count; INR, international normalized ratio. | | | | |

**Supplementary table 2. Baseline characteristics of medium- and high-risk stratifications**

| **Baseline characteristics** | **Total patients** | **Cohorts before PSM** | | **Cohort of PS0 after PSM ^$^** |
| --- | --- | --- | --- | --- |
|  |  | **PS 0** | **PS 1** |  |
| Medium-risk stratification, n | 612 | 462 | 150 | 150 |
| Age, <70 / ≥70, n (%) | 553 (90.4) /59 (79.6) | 419 (90.7) / 43 (9.3) | 134 (89.3) / 16 (10.7) | 134 (89.3) / 16 (10.7) |
| Gender, male / female, n (%) | 505 (82.5) / 107 (17.5) | 379 (82.0) / 83 (18.0) | 126 (84.0) / 24 (16.0) | 127 (84.7) / 23 (15.3) |
| Etiology, HBV (+) / HBV (-)，n (%) | 561 (91.7) / 51 (8.3) | 427 (92.4) / 35 (7.6) | 134 (89.3) / 16 (10.7) | 139 (92.7) / 11 (7.3) |
| Child-pugh score, 5 / 6, n (%) | 552 (90.2) / 60 (9.8) | 409 (88.5) / 53 (11.5) | 143 (95.3) / 7 (4.7) | 141 (94.0) / 9 (6.0) |
| AFP, <400 / ≥400 ng/ml, n (%) ^*^ | 391 (63.9) / 221 (36.1) | 280 (60.6) / 182 (39.4) | 111 (74.0) / 39 (26.0) | 98 (65.3) / 52 (34.7) |
| Tumor number, 1 / >1, n (%) | 597 (97.5) / 15 (2.5) | 450 (97.4) / 12 (2.6) | 147 (98.0) / 3 (3.7) | 150 (100.0) / 0 (0.0) |
| Tumor size, ≤5 / >5 cm, n (%) ^*^ | 273 (44.6) / 339 (55.4) | 228 (49.4) / 234 (50.6) | 45 (30.0) / 105 (70.0) | 57 (38.0) / 93 (62.0) |
| Albumin, >35 / ≤35 g/L, n (%) | 560 (91.5) / 52 (8.5) | 416 (90.0) / 46 (10.0) | 144 (96.0) / 6 (9.0) | 145 (96.7) / 5 (3.3) |
| TBIL, ≤17.1 / >17.1 μmol/L, n (%) | 384 (62.7) / 228 (37.3) | 289 (62.6) / 173 (37.4) | 95 (63.3) / 55 (36.7) | 94 (62.7) / 56 (37.3) |
| AST, U/L, median (IQR) ^*^ | 35.3 (27.2-52.0) | 35.0 (27.0-49.1) | 42.2 (28.4-59.3) | 35.0 (28.4-59.3) |
| ALT, U/L, median (IQR) | 33.0 (23.1-52.0) | 32.0 (23.9-51.0) | 35.0 (23.0-62.0) | 32.0 (24.0-51.0) |
| WBC, ×10^9^ /L, median (IQR) | 5.3 (4.2-6.7) | 5.3 (4.2-6.8) | 5.7 (4.3-7.2) | 5.4 (4.4-6.7) |
| BUN, mmol/L, median (IQR) | 5.4 (4.5-6.4) | 5.5 (4.6-6.4) | 5.4 (4.5-6.5) | 5.3 (4.4-6.1) |
| PLT, ×109 /L, median (IQR)^* $^ | 151.0 (110.0-196.8) | 149.0 (108.8-193.0) | 155.0 (112.8-211.0) | 152.5 (109.5-199.0) |
| INR, median (IQR) | 1.02 (0.97-1.07) | 1.02 (0.97-1.08) | 1.02 (0.95-1.07) | 1.01 (0.97-1.07) |
| High-risk stratification, n | 257 | 175 | 82 | 82 |
| Age, <70 / ≥70, n (%) | 245 (95.3) / 12 (4.7) | 167 (95.4) / 8 (4.6) | 78 (95.1) / 4 (4.9) | 79 (96.3) / 3 (3.7) |
| Gender, male / female, n (%) | 213 (82.9) / 44 (17.1) | 146 (83.4) / 29 (16.6) | 67 (81.7) / 15 (18.3) | 70 (68.5) / 12 (13.5) |
| Etiology, HBV (+) / HBV (-)，n (%) | 244 (94.9) / 13 (5.1) | 167 (95.4) / 8 (4.6) | 77 (93.9) / 5 (4.1) | 79 (96.3) / 3 (3.7) |
| Child-pugh score, 5 / 6, n (%) | 200 (77.8) / 57 (22.2) | 200 (77.8) / 57 (22.2) | 134 (76.6) / 41 (23.4) | 68 (82.9) / 14 (17.1) |
| AFP, <400 / ≥400 ng/ml, n (%) | 32 (12.5) / 225 (87.5) | 22 (12.6) / 153 (87.4) | 10 (10.2) / 72 (87.8) | 7 (8.5) / 75 (91.5) |
| Tumor number, 1 / >1, n (%) | 256 (99.6) / 1 (0.4) | 174 (99.4) / 1 (0.6) | 82 (100.0) / 0 (0.0) | 82 (100.0) / 0 (0.0) |
| Tumor size, ≤5 / >5 cm, n (%) | 12 (4.7) / 245 (95.3) | 10 (5.7) / 165 (94.3) | 2 (2.4) / 80 (97.6) | 3 (3.7) / 79 (96.3) |
| Albumin, >35 / ≤35 g/L, n (%) | 200 (77.8) / 57 (22.2) | 134 (76.6) / 41 (23.4) | 66 (80.5) /16 (19.5) | 68 (82.9) / 14 (17.1) |
| TBIL, ≤17.1 / >17.1 μmol/L, n (%) | 166 (64.6) / 91 (35.4) | 107 (61.1) / 68 (38.9) | 59 (72.0) / 23 (28.0) | 54 (65.9) / 28 (34.1) |
| AST, U/L, median (IQR) | 44.0 (29.5-69.5) | 44.3 (29.5-67.0) | 30.0 (44.3-70.0) | 40.5 (28.0-72.0) |
| ALT, U/L, median (IQR) | 35.0 (23.0-60.0) | 35.0 (24.0-64.3) | 35.0 (22.0-53.0) | 35.0 (23.0-63.0) |
| WBC, ×10^9^ /L, median (IQR) | 5.7 (4.7-7.0) | 5.7 (4.6-7.0) | 5.8 (4.8-7.2) | 5.6 (4.7-6.7) |
| BUN, mmol/L, median (IQR) ^$^ | 5.0 (4.0-6.2) | 5.2 (4.2-6.1) | 4.5 (3.6-6.2) | 5.3 (4.5-6.3) |
| PLT, ×10^9^ /L, median (IQR) ^*^ ^$^ | 173.0 (124.0-222.0) | 162.0 (118.0-211.5) | 161.0 (191.0-241.0) | 167.0 (125.0-214.0) |
| INR, median (IQR) | 1.02 (0.97-1.07) | 1.02 (0.97-1.08) | 1.02 (0.98-1.07) | 1.02 (0.98-1.07) |
| ^*^ Variables with significant difference between PS 0 and PS 1 cohorts (P＜0.05).  ^$^ Variables with significant difference between PS 0 and PS 1 cohorts (P＜0.05) after a 1:1 propensity score matching (PSM; caliper value of 0.2).  Abbreviations: HCC, hepatocellular carcinoma; PS, performance status; HBV, hepatitis B virus; AFP, alpha fetoprotein; TBIL, total bilirubin; AST, aspartate aminotransferase; ALT, alamine aminotransferase; WBC, white blood cell; BUN, blood urea nitrogen; PLT, platelet count; INR, international normalized ratio. | | | | |

**Supplementary table 3. The prognostic value of PS in different stratifications**

| **Risk stratification** | **Patients, n (%)** | | **Univariate analysis** | | **Multivariate analysis** | | **Variables included in multivariate analyses ^*^** |
| --- | --- | --- | --- | --- | --- | --- | --- |
|  | **PS0** | **PS1** | **HR (95%CI)** | **P value** | **HR (95%CI)** | **P value** |  |
| Tumor burden ^**^ | | | | | | | |
| Less than 7 | 846 (78.8) | 228 (21.2) | 0.59 (0.44-0.79) | <0.001 | 0.61 (0.46-0.82) | 0.010 | AFP, ALBI-grade, WBC, PLT, INR |
| 7 to 11 | 266 (76.9) | 80 (23.1) | 0.45 (0.29-0.70) | <0.001 | 0.49 (0.32-0.76) | 0.002 | AFP, ALBI-grade, AST |
| Beyond 11 | 81 (70.4) | 34 (29.6) | 0.42 (0.21-0.81) | 0.010 | 0.41 (0.21-0.79) | 0.008 | AFP |
| ALBI grade | | | | | | | |
| Grade1 | 873 (79.0) | 223 (21.0) | 0.53 (0.37-0.76) | <0.001 | 0.51 (0.38-0.69) | <0.001 | AFP, Tumor burden, WBC, PLT, INR |
| Grade2 | 356 (74.9) | 119 (25.1) | 0.52 (0.39-0.71) | <0.001 | 0.56 (0.39-0.80) | 0.002 | AFP, BUN |
| Grade3 | 0 (0.0) | 0 (0.0) | - | - | - | - | - |
| AFP | | | | | | | |
| <400 ng/ml | 858 (78.8) | 231 (21.2) | 0.55 (0.41-0.74) | <0.001 | 0.55 (0.41-0.74) | <0.001 | ALBI-grade, PLT, INR |
| ≥400 ng/ml | 335 (75.1) | 111 (24.9) | 0.52 (0.37-0.73) | <0.002 | 0.52 (0.36-0.74) | <0.001 | Tumor burden, WBC, PLT, AST |
| ^*^ Variables with statistically significant differences in univariate analysis and included in multivariate analysis for adjustment.  ^**^ Tumor burden = maximum tumor diameter (cm) + number of tumors  Abbreviations: HR, hazard ratio; PS, performance status; AFP, alpha fetoprotein; AST, aspartate aminotransferase; WBC, white blood cell; BUN, blood urea nitrogen; PLT, platelet count; INR, international normalized ratio; ALBI-grade: the Albumin-Bilirubin grade. | | | | | | | |
